# A Potential Role for Endogenous Glucagon in Preventing Post-Bariatric Hypoglycemia

**DOI:** 10.3389/fendo.2020.608248

**Published:** 2020-11-30

**Authors:** Carolina B. Lobato, Sofia S. Pereira, Marta Guimarães, Bolette Hartmann, Nicolai J. Wewer Albrechtsen, Linda Hilsted, Jens J. Holst, Mário Nora, Mariana P. Monteiro

**Affiliations:** ^1^ Endocrine, Cardiovascular & Metabolic Research, Unit for Multidisciplinary Research in Biomedicine (UMIB), University of Porto, Porto, Portugal; ^2^ Department of Anatomy, Institute of Biomedical Sciences Abel Salazar (ICBAS), University of Porto, Porto, Portugal; ^3^ Instituto de Investigação e Inovação em Saúde (I3S), Universidade do Porto, Porto, Portugal; ^4^ Institute of Molecular Pathology and Immunology of the University of Porto (IPATIMUP), Porto, Portugal; ^5^ Department of General Surgery, Centro Hospitalar de Entre o Douro e Vouga, Santa Maria da Feira, Portugal; ^6^ Department of Biomedical Sciences, Faculty of Health and Medical Sciences, University of Copenhagen, Copenhagen, Denmark; ^7^ Novo Nordisk Foundation Center for Basic Metabolic Research, Faculty of Health and Medical Sciences, University of Copenhagen, Copenhagen, Denmark; ^8^ Department of Clinical Biochemistry, Rigshospitalet, University of Copenhagen, Copenhagen, Denmark

**Keywords:** glucagon, glucagon-like peptide-1, hyperinsulinemia, hypoglycemia, Roux-en-Y gastric bypass

## Abstract

Obesity and obesity-related diseases are major public health concerns that have been exponentially growing in the last decades. Bariatric surgery is an effective long-term treatment to achieve weight loss and obesity comorbidity remission. Post-bariatric hypoglycemia (PBH) is a late complication of bariatric surgery most commonly reported after Roux-en-Y gastric bypass (RYGB). PBH is the end result of postprandial hyperinsulinemia but additional endocrine mechanisms involved are still under debate. Our aim was to characterize entero-pancreatic hormone dynamics associated with postprandial hypoglycemia after RYGB. Individuals previously submitted to RYGB (*N*=23) in a single tertiary hospital presenting PBH symptoms (*Sym*, *n*=14) and asymptomatic weight-matched controls (*Asy*, *n*=9) were enrolled. Participants underwent a mixed-meal tolerance test (MMTT) to assess glucose, total amino acids (total AA), insulin, C-peptide, glucagon, glucose-dependent insulinotropic polypeptide (GIP), glucagon-like peptide-1 (GLP-1), and neurotensin (NT). We found that hypoglycemia during the MMTT was equally frequent in *Sym* and *Asy* groups (*p*=1.000). Re-grouped according to glucose nadir during the MMTT (*Hypo* *n*=11 vs *NoHypo* *n*=12; nadir <3.05 mmol/l vs ≥3.05 mmol/l), subjects presented no differences in anthropometric (BMI: *p*=0.527) or metabolic features (HbA_1c_: *p*=0.358), yet distinct meal-elicited hormone dynamics were identified. Postprandial glucose excursion and peak glucose levels were similar (*p*>0.05), despite distinct late glycemic outcomes (t=60 min and t=90 min: *p*<0.01), with overall greater glycemic variability in *Hypo* group (minimum-to-maximum glucose ratio: *p*<0.001). *Hypo* group meal-triggered hormone profile was characterized by lower early glucagon (t=15 min: *p*<0.01) and higher insulin (t=30 min: *p*<0.05, t=45 min: *p*<0.001), C-peptide (t=30 min: *p*<0.01, t=45 min: *p*<0.001, t=60 min: *p*<0.05), and GLP-1 (t=45 min: *p*<0.05) levels. Hyperinsulinemia was an independent risk factor for hypoglycemia (*p*<0.05). After adjusting for hyperinsulinemia, early glucagon correlated with glycemic nadir (*p*<0.01), and prevented postprandial hypoglycemia (*p*<0.05). A higher insulin to glucagon balance in *Hypo* was observed (*p*<0.05). No differences were observed in total AA, GIP or NT excursions (*p*>0.05). In sum, after RYGB, postprandial hyperinsulinemia is key in triggering PBH, but a parallel and earlier rise in endogenous glucagon might sustain the inter-individual variability in glycemic outcome beyond the effect of hyperinsulinism, advocating a potential pivotal role for glucagon in preventing hyperinsulinemic hypoglycemia.

## Introduction

Bariatric surgery is the most effective long-term treatment for obesity and related disorders (1). Despite the health benefits from weight loss surgical interventions, post-bariatric patients management is essential to avoid nutrient deficiencies as well as for timely detection of other less frequent surgical or medical complications (1).

Meal-triggered hypoglycemia can occur after several upper gastrointestinal surgical procedures (2, 3). A reduction in postprandial glucose nadir has also been reported after non-surgical weight loss interventions (4–6). Post-bariatric hypoglycemia (PBH) is a late complication of bariatric surgery (7), for which there is a lack of consensus on diagnostic criteria, since etiology is still a matter of debate (2).

Postprandial hyperinsulinemia is a common finding and considered to be the ultimate trigger of postprandial hypoglycemia (7). However, the leading cause for the hyperinsulinemia observed remains to be elucidated. Structural pancreatic changes, such as nesidioblastosis, insulinoma or other insulinotropic neuroendocrine tumors, responsible for autonomous hyperinsulinemia are rarely found to be the cause (8), although these entities must be ruled out before assuming PBH diagnosis (9). Alternatively, altered gut hormone dynamics (10, 11) or lack of putative unidentified “anti-incretin” factors (12) were also hypothesized to have a role in triggering the hyperinsulinemic response observed in PBH.

The fact that PBH frequency can be mitigated by dietary interventions (2, 13, 14) and pharmacological interventions that reduce carbohydrate digestion or absorption, delay gastro-intestinal transit time, limit insulin secretion or suppress incretin effects (10, 15, 16), suggests a role for entero-pancreatic hormone dynamics in this condition.

Thus, the goal of this study was to characterize the entero-pancreatic hormone dynamics associated with PBH in patients previously submitted to Roux-en-Y gastric bypass (RYGB).

## Materials and Methods

### Patient Selection

Participants were recruited from our single center cohort of post-bariatric patients submitted to RYGB by the same surgical team using a standardized technical procedure as previously described (17). Patients enrolled in the study (*N*=23) included subjects that spontaneously reported autonomic and neuroglycopenic symptoms suggestive of PBH, (*Sym*, *n*=14; reported as “Self-reported hypoglycemia symptoms” in **Table 1**), matched to asymptomatic surgical controls (*Asy*, *n*=9) recruited from the patient cohort under routine follow-up at our center. Reports of sweating, tremor, palpitations, anxiety, hunger, or paresthesia/tingling were recognized as autonomic symptoms, while headache, slurred speech, drowsiness, weakness, visual disturbances, concentration difficulties, confusion, seizures, or altered consciousness were assumed as neuroglycopenic symptoms (2). All participants reporting consistently at least one of the previous symptoms at enrolment were allocated into the *Sym* group. Inclusion criteria comprised previous RYGB surgery, being weight stable—defined as less than 10% body weight variation over the previous 6 months—and having an HbA1c <6.5% and fasting plasma glucose <7.0 mmol/l at the time of screening visit. Exclusion criteria were current pregnancy, taking glucose-lowering drugs or prior diagnosis of any medical condition that could be responsible for hypoglycemia after comprehensive workout.

**Table 1:** Anthropometric, demographic and metabolic features of post-RYGB patients according to glucose profile during the MMTT (*Hypo* and *NoHypo*).

	MMTT result		
	Hypo	NoHypo	p value
**N** (% of total)	11 (47.8%)	12 (52.2%)	NA
**Sex** (male/female)	2/9	2/10	1.000
**Age at surgery** (years)	40.6 (32.5-48.5)	45.2 (39.0-47.8)	0.294
**History of T2DM before surgery** (yes/no)	0/11	3/9	0.217
**Follow-up time after surgery** (years)	5.1 ± 0.6	3.8 ± 0.7	0.159
**BMI before surgery** (kg/m^2^)	40.1 ± 1.5	41.5 ± 1.6	0.527
**BMI after surgery** (kg/m^2^)	27.6 ± 0.7	28.5 ± 1.0	0.502
**%EBMIL** (%)	83.2 ± 4.0	81.9 ± 6.1	0.864
**%TWL** (%)	30.6 ± 2.1	31.0 ± 2.1	0.884
**HbA1c** (mmol/mol)	34.4 ± 1.3	35.8 ± 1.0	0.365
**HbA1c** (%)	5.3 ± 0.1	5.4 ± 0.1	0.358
**HOMA2-B** (%)	77.9 ± 6.7	70.1 ± 5.6	0.376
**HOMA2-S** (%)	144.2 ± 11.6	152.3 ± 15.6	0.685
**HOMA2-IR**	0.8 (0.6-0.8)	0.6 (0.5-1.0)	0.618
**Self-reported hypoglycemia symptoms** (yes/no)	7/4	7/5	1.000
**Dumping criteria** (yes/no)	11/0	10/2	0.478

Results are presented as mean ± SEM, median (interquartile range) or proportions. MMTT, mixed-meal tolerance test; Hypo, individuals developing a glucose nadir <3.05 mmol/l during the MMTT; NoHypo, subjects with glucose levels ≥3.05 mmol/l during MMTT; T2DM, type 2 diabetes mellitus; BMI, body mass index; EBMIL, excess BMI loss; TWL, total weight loss; HOMA2-B, homeostasis model assessment for β-cell function; HOMA2-S, homeostasis model assessment for insulin sensitivity; HOMA2-IR, homeostasis model assessment for insulin resistance.

The study protocol was reviewed and approved Local Institutional Ethics Committee (Comissão de Ética para a Saúde CHEDV, Epe). Patients provided their written informed consent to participate in this study. No potentially identifiable human images or data is presented in this study.

### Study Design

After an overnight 12-h fast, patients underwent a mixed-meal tolerance test (MMTT) with a standardized liquid meal (Fresubin Energy Drink, 200 ml, 300 kcal [50E% carbohydrate, 15E% protein and 35E% fat]; Fresenius Kabi Deutschland, Bad Homburg, Germany), based on macronutrient composition in accordance to post-bariatric surgery nutritional recommendations, as well as to allow comparisons with previous studies (15, 17). Patients were instructed to abstain from alcohol consumption and strenuous physical activity the day before trial-days. Patients were requested to slowly drink the liquid meal over the first 15 min of the MMTT (grey shade in Figures), to assure consistency among subjects.

Peripheral venous blood sampling was performed using EDTA tubes (S-Monovette^®^ 9.0 ml, K2 EDTA Gel, 1.6 mg/ml, Sarstedt) at pre-established timepoints before and after the start of meal ingestion (baseline and 15, 30, 45, 60, 90, and 120 min), with simultaneous monitoring of pulse and blood pressure (BP). Samples were kept refrigerated until separation and plasma was stored at -20°C until analyzed.

### Study Groups

Study subjects were recruited and initially grouped according to symptomatic status at presentation (*Sym*, *n*=14 and *Asy*, *n*=9). Subjects were then re-allocated into two different groups according to the glucose profile during the MMTT, into an *Hypo* (*n*=11) group, including patients with a glycemic nadir <3.05 mmol/l (<55 mg/dl) during the MMTT, and a *NoHypo* (*n*=12) group, comprising the participants with glucose ≥3.05 mmol/l (≥55 mg/dl) during the entire test.

### Biochemical Measurements

Whole blood glucose was assessed using a commercially available glucometer (Freestyle Precision Neo Glucose meter, Abbott, USA). Plasma insulin and C-peptide levels were measured by electrochemiluminescence sandwich immunoassay (ECLIA) (Cobas 8000, model e602, Roche Diagnostics, USA), against two liquid human serum-based controls: Liquichek™ Immunoassay Plus Control, Level 1 #361 and Level 3 #363, Bio-Rad for insulin and Liquichek™ Specialty Immunoassay Control, Level 1 #364 Level 3 #366, Bio-Rad for C-peptide. Other plasma hormone levels were quantified by radioimmunoassay (RIA), using analytical methods previously described (17), namely glucagon, with no cross-reactivity with GLP-1, glicentin, or oxyntomodulin (antibody code no 4305) (18), total glucose-dependent insulinotropic polypeptide (GIP) (antibody code no 867) (19), total glucagon-like peptide-1 (GLP-1) (antibody code no 89390) (20) and neurotensin (NT) (antibody code no 3D97) (21). Sensitivity for all assays was below 1 pmol/l and intra-assay coefficient of variation below 10%. Total amino acids (total AA) were assayed as previously described (22) in plasma samples from 19 subjects (*Hypo* *n*=8 and *NoHypo* *n*=11).

### Calculations

Percentage of total weight loss (%TWL) and of excess body mass index (BMI) loss (%EBMIL) were determined, respectively, as [(preoperative weight – weight at MMTT) ÷ (weight at MMTT) x 100] and [(preoperative BMI – BMI at MMTT) ÷ (preoperative BMI - 25) × 100], with 25kg/m^2^ as target BMI.

Updated homeostasis model assessment indexes (HOMA2) were determined using the HOMA Calculator version 2.2.3 (http://www.dtu.ox.ac.uk, accessed April 2018) as surrogate measures of beta cell function (HOMA2-B) and peripheral insulin sensitivity (HOMA2-S) and resistance (HOMA2-IR).

A pulse rise greater than 10 beats per minute during the first 30 min of the provocative test was assumed to be a sensitive and specific early dumping sign (23) and referred to as “Dumping criteria”.

Incremental area under the curve (iAUC) was calculated using the trapezoidal rule, with deduction of the fasting hormonal levels from the subsequent time points. To assess glycemic variability, minimum-to-maximum glucose ratio (MMGR) was calculated as the ratio from maximum to minimum glycemic values observed during the MMTT. Insulinogenic index (IGI) was calculated by the ratio of incremental C-peptide from fasting to 30 min of the MMTT to glycemia variation in the same time window. Oral glucose insulin sensitivity (OGIS) was determined (24, 25) and multiplied with IGI to calculate the Disposition Index, as a measure of insulin secretion adjusted for insulin sensitivity. Insulin secretion rate (ISR) was obtained from C-peptide plasma levels (CV 5%) with adjustment for sex, age and BMI by ISEC program (26). Insulin clearance was then retrieved from tAUC_ISR_/tAUC_insulin_. Insulin:glucagon ratio (IGR) was used to assess the variance between catabolic and anabolic responses to the meal-stimulus (27). A gut hormone incretin/glucagon ratio was post-hoc computed as the product of GLP-1 and GIP divided by glucagon levels (GLP-1*GIP/Glucagon).

### Statistical Analysis

Statistical analysis was performed using the GraphPad Prism version 8.0.1 for Windows (GraphPad Software, La Jolla California USA). Correlations and logistic regressions were performed with IBM^®^ SPSS^®^ Statistics version 25 for Windows. Differences between the two groups were considered statistically significant when *p* value was below 0.05.

Normality of continuous variables was assessed using the D’Agostino & Pearson test. For normally distributed variables, the two groups were compared using unpaired two-tailed t-test and results are presented as mean ± standard error of the mean (SEM). Variables that do not follow a normal distribution are represented as median (interquartile range) and were compared using Mann-Whitney test. To assess dynamic changes during the MMTT, changes of hormones and metabolites between time points were compared using a two-way ANOVA with Sidak’s *post hoc* test. Categorical variables are expressed as proportions and were compared by Fisher’s exact test.

Preliminary bivariate Spearman’s rho correlations were performed between postprandial hormonal excursions (glucagon, insulin, GLP-1, GIP, and NT) and glycemic nadir during MMTT (not-Gaussian distributed). For variables that correlated with the glycemic nadir (*p*<0.05), partial correlations and binary logistic regressions were performed to assess whether these correlations were independent and to disclose the potential of hormone excursions to predict hypoglycemia respectively.

## Results

### Subject Anthropometric and Clinical Features

Subject groups initially enrolled on the basis of self-reported symptoms suggestive of PBH (*Sym*, *n*=14) or as asymptomatic controls (*Asy*, *n*=9) presented no significant differences in anthropometric, demographic and metabolic features (**Supplementary Table 1**). Glycemic profiles (iAUC_0’-120’_: *p*=0.214) during the MMTT were not significantly different between the two participants groups. In addition, the frequency of postprandial hypoglycemia during the MMTT was similar in two groups, with 50.0% (7 of 14) of the *Sym* and 44.4% (4 of 9) of the *Asy* individuals developing hypoglycemia (*p*=1.000, **Supplementary Table 1**).

Based on the glycemic response during the MMTT, study participants were then re-grouped into *Hypo* group (glucose nadir <3.05 mmol/l, *n*=11) or *NoHypo* (glucose ≥3.05 mmol/l during MMTT, *n*=12).

### Demographic Data

There were no differences in anthropometric or demographic features between *Hypo* and *NoHypo* subjects. Three individuals had been diagnosed with type 2 diabetes (T2DM) prior to RYGB but were in remission and off any glucose lowering medications at study entry (HbA1c = 5.2%, 5.9%, and 5.5%, 2.1, 2.6, and 8.2 years after RYGB, respectively). None of the patients with T2DM prior to RYGB presented glucose nadir <3.05 mmol/l during the MMTT (*NoHypo*) (**Table 1**).

Comparing biochemical profiles of *Hypo* and *NoHypo* groups, there were no differences in fasting glucose, HbA1c levels and surrogate measures for beta cell function (HOMA2-B), hepatic insulin sensitivity (HOMA2-S) and peripheral insulin resistance (HOMA2-IR) (*p*>0.05, **Table 1**), which were also within the normal physiological intervals (28).

### Glucose, Total Amino Acids, and Hormone Profiles During MMTT

**Figure 1:**
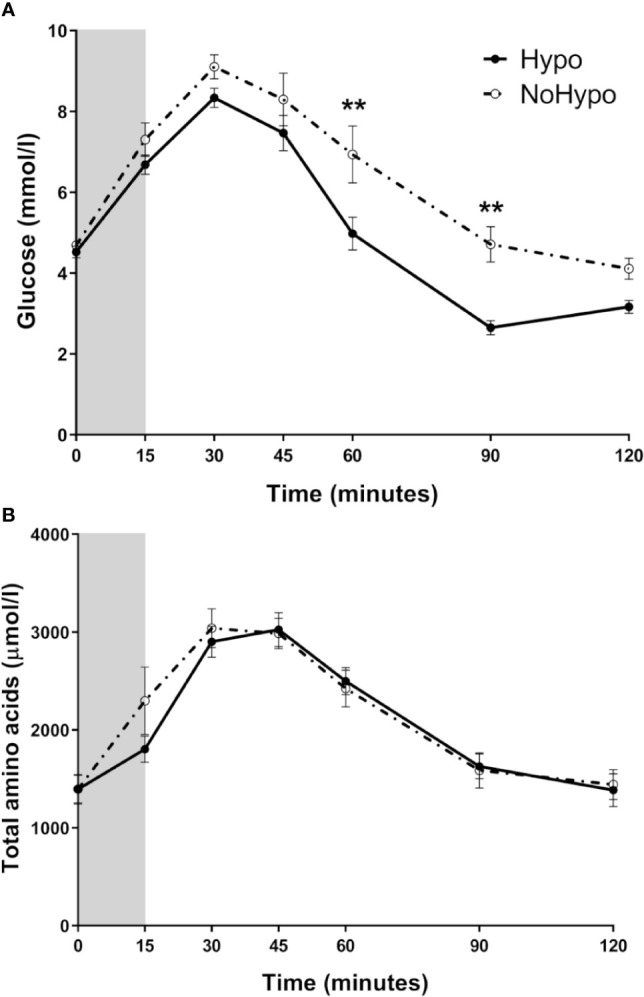
Peripheral levels of glucose **(A)** and total amino acids **(B)**, in post-RYGB patients according to the MMTT response (*Hypo* and *NoHypo*). Data is presented as mean ± SEM. Statistically significant differences are marked as **p < 0.01.

*Hypo* and *NoHypo* groups presented distinctive glycemic profiles, particularly at 60 and 90 min of the MMTT (*p*=0.003 and *p*=0.002 respectively), with *Hypo* group presenting a glucose peak (*p*=0.049) and nadir (*p*<0.001) 11% and 38% lower, respectively. *Hypo* subjects also presented a 40% higher glycemic variability (MMGR: *p*<0.001) (**Table 2**, **Figure 1A**). Total AA excursion during MMTT was not significantly different in the two study groups (*p*>0.05) (**Table 2**, **Figure 1B**). No differences in vasoactive response, as assessed by pulse rate and BP curves, were observed (*p*>0.05, data not shown).

**Table 2:** Meal induced glucose and hormone responses in post-RYGB patients according to MMTT glycemic response (*Hypo* and *NoHypo*).

	MMTT result		
	*Hypo*	*NoHypo*	*p* value
**N (% of total)**	11 (47.83)	12 (52.17%)	NA
**Glucose**			
Fasting (mmol/l)	4.5 ± 0.1	4.7 ± 0.3	0.324
iAUC_0’-120’_ (mmol/l x min)	223.5 ± 13.2	263.7 ± 27.5	0.214
Peak (mmol/l)	8.5 ± 0.3	9.6 ± 0.4	**0.049**
Nadir (mmol/l)	2.5 ± 0.1	4.0 ± 0.3	**<0.001**
MMGR	3.5 ± 0.2	2.5 ± 0.1	**<0.001**
**Total AA**			
Fasting (µmol/l)	1394 ± 151.7	1396 ± 141.5	0.992
iAUC_0’-120’_ (µmol/l x min)	92400 ± 16433	95334 ± 12932	0.889
**Insulin**			
Fasting (pmol/l)	40.9 (29.4–45.7)	32.8 (27.2–52.9)	0.576
iAUC_0’-120’_ (nmol/l x min)	99.1 (60.8–113.8)	45.4 (37.4–53.6)	**0.019**
IGI	44.4 (32.8–54.6)	24.9 (16.6–36.2)	**0.007**
OGIS [ml/(min x m^2^)]	395.9 (380.3–448.1)	401.6 (376.6–424.2)	0.695
Disposition Index	17646.2 ± 1385.6	10848.6 ± 1630.5	**0.005**
**C-peptide**			
Fasting (pmol/l)	492.3 (435.5–602.9)	505.0 (375.1–544.8)	0.910
iAUC_0’-120’_ (nmol/l x min)	235.7 ± 17.2	168.4 ± 15.3	**0.008**
**ISR**			
Fasting [pmol/(kg.min)]	1.7 (1.5–2.0)	1.6 (1.3–1.8)	0.518
iAUC_0’-120’_ (pmol/kg)	1016 ± 67.3	694.2 ± 60.3	**0.002**
ISR_iAUC_/Glucose_iAUC_	323.5 ± 23.7	226.3 ± 34.8	**0.034**
**Insulin Clearance**			
Fasting	3.0 ± 0.2	3.4 ± 0.3	0.369
Postprandial_0’-120’_	0.8 (0.7–1.0)	1.2 (0.8–1.7)	0.079
**Glucagon**			
Fasting (pmol/l)	5.8 ± 0.8	6.3 ± 0.8	0.695
iAUC_0’-120’_ (pmol/l x min)	869.3 ± 121.3	758.3 ± 124.8	0.532
**GLP-1**			
Fasting (pmol/l)	21.1 ± 2.5	14.8 ± 2.4	0.080
iAUC_0’-120’_ (pmol/l x min)	7717 ± 958.1	6558 ± 725.3	0.341
**GIP**			
Fasting (pmol/l)	4.0 (3.0–6.0)	6.0 (2.6–9.0)	0.384
iAUC_0’-120’_ (pmol/l x min)	4298 (3353–5689)	3683 (2607–5665)	0.339
**Neurotensin**			
Fasting (pmol/l)	20.0 (18.0–24.0)	20.0 (20.0–27.0)	0.653
iAUC_0’-120’_ (pmol/l x min)	13440 (8940–20100)	12765 (10774–18764)	0.833

Results are presented as mean ± SEM or median (interquartile range). Statistically significant differences (p value <0.05) are highlighted in bold. MMTT, mixed-meal tolerance test; Hypo, individuals developing a glucose nadir <3.05 mmol/l during the MMTT; NoHypo, subjects with glucose levels ≥3.05 mmol/l during MMTT; MMGR, minimum-to-maximum glucose ratio; Total AA, total amino acids; IGI, insulinogenic index; OGIS, oral glucose insulin sensitivity; iAUC, postprandial incremental AUC; ISR, insulin secretion rate; GIP, glucose-dependent insulinotropic polypeptide; GLP-1, glucagon-like peptide-1.

**Figure 2:**
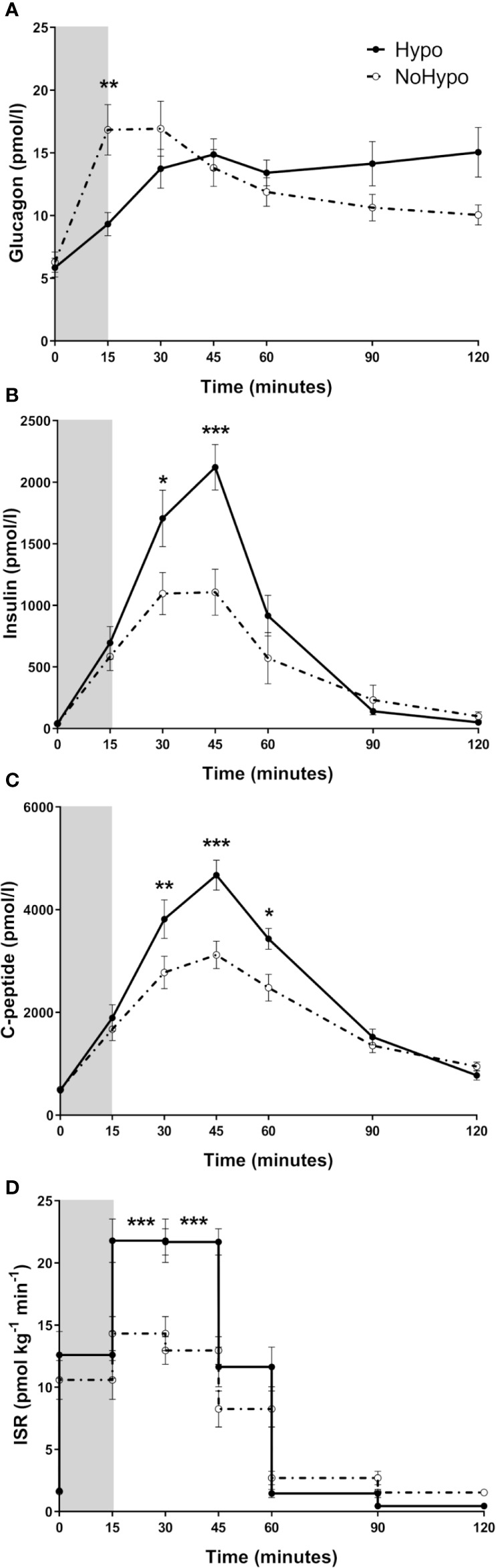
Circulating levels of glucagon **(A)**, insulin **(B)**, C-peptide **(C),** and ISR **(D)**, in post-RYGB patients grouped according to MMTT response (*Hypo* and *NoHypo*). Data is presented as mean ± SEM. Statistically significant differences are marked as *p < 0.05, **p < 0.01, and ***p < 0.001. ISR, Insulin secretion rate.

*Hypo* and *NoHypo* groups depicted significantly different hormone profiles along the MMTT, despite no significant differences at baseline were observed. Insulin and co-secreted C-peptide postprandial levels were significantly higher in *Hypo* group (insulin t=30min: *p*=0.015 and t=45min: *p*<0.001; C-peptide t=30min: *p*=0.008, t=45min: *p*<0.001, and t=60min: *p*=0.018) and throughout the MMTT (iAUC: *p*=0.019), yielding a significantly higher Disposition Index (*p*=0.005), IGI (*p*=0.007), and ISR (iAUC_0’-120’_: *p*=0.002; 15–30 and 30–45 min: *p*<0.001), even when adjusted for glucose excursion (ISR_iAUC_/Glucose_iAUC_: *p*=0.034) (**Table 2**, **Figures 2B–D**). Hyperinsulinemia (insulin iAUC_0’-45’_), with plasma insulin peak levels twice higher in the *Hypo* group (**Figure 2B**), was a risk for hypoglycemia during MMTT (binary logistic regression: *p*=0.042). Insulin excursion (iAUC_0’-45’_) was inversely correlated with glycemic nadir even when adjusted for the effect of early glucagon excursion (partial correlation: *r*=-0.475, *p*=0.026).

In *NoHypo* group, peak glucagon levels occurred earlier and were significantly higher when compared to *Hypo* ones (15 min: *p*=0.002, **Figure 2A**). Early glucagon excursion (glucagon levels at 15 min time point of the MMTT) was associated with the risk of later hypoglycemia (binary logistic regression: *p*=0.045) and correlated positively with glycemic nadir, even after suppressing postprandial hyperinsulinemia effect (partial correlation: *r*=0.628, *p*=0.002).

**Figure 3:**
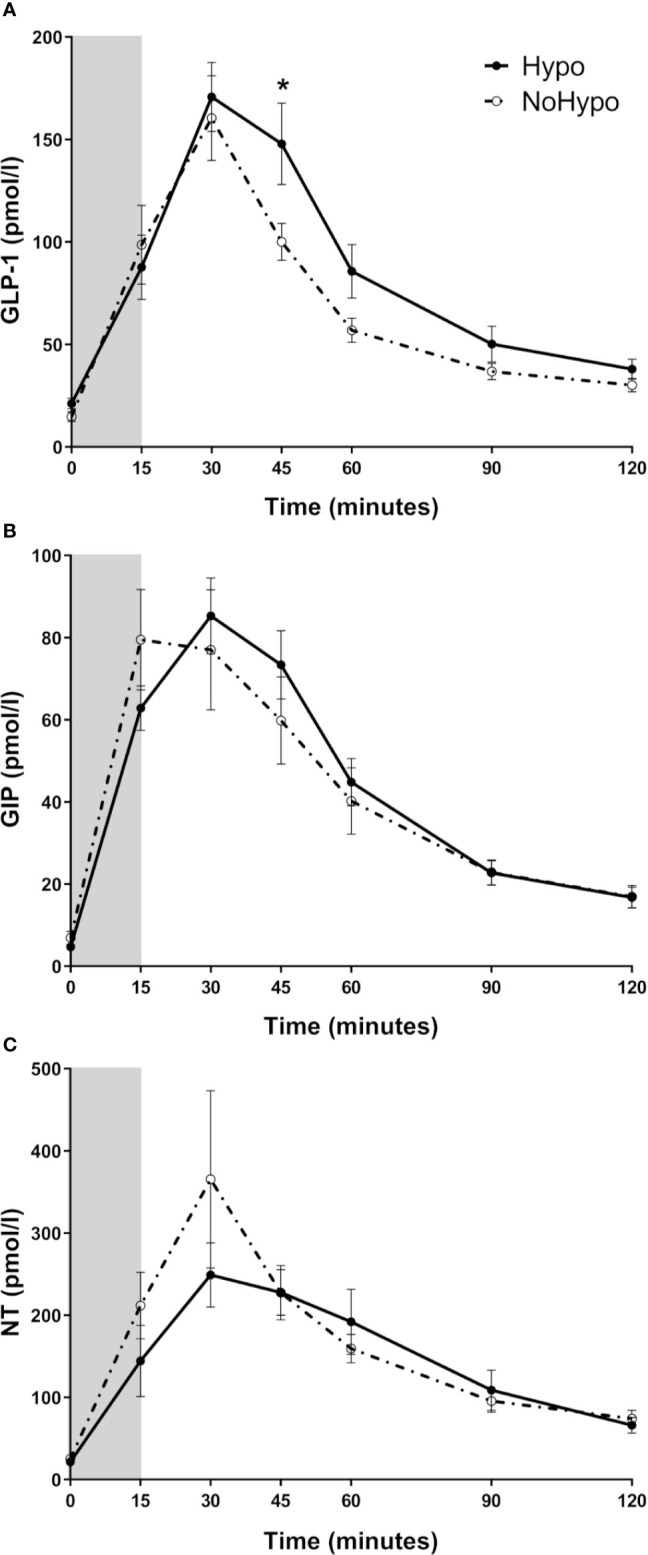
Plasma levels of GLP-1 **(A)**, GIP **(B),** and NT **(C)**, in post-RYGB patients according to the MMTT response (*Hypo* and *NoHypo*). Data is presented as mean ± SEM. Statistically significant differences are marked as *p < 0.05. GIP, glucose-dependent insulinotropic polypeptide; GLP-1, glucagon-like peptide-1; NT, Neurotensin.

Higher post-peak GLP-1 levels were observed in *Hypo* when compared to *NoHypo* group (45 min: *p*=0.049, **Figure 3A**). There were no significant differences in GIP (**Figure 3B**) and NT (**Figure 3C**) profiles between the study groups.

**Figure 4:**
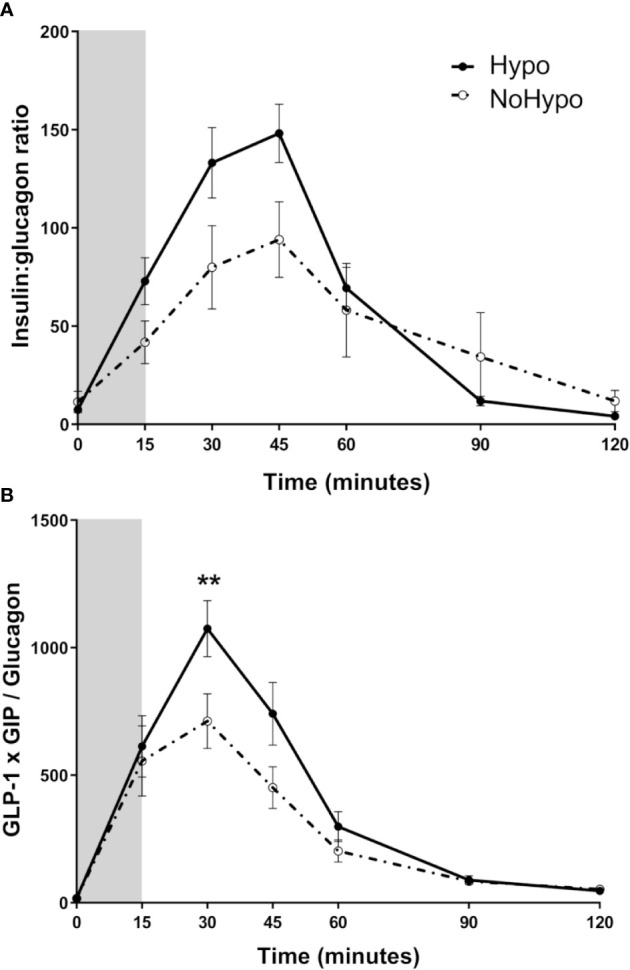
Insulin:glucagon ratio **(A)** and incretin:glucagon ratio (GLP-1*GIP/Glucagon) **(B)** combining the two major incretin hormones (GLP-1 and GIP), in post-RYGB patients according to the MMTT response (*Hypo* and *NoHypo*). Data is presented as mean ± SEM. Statistically significant differences are marked as **p < 0.01. Abbreviations: GIP, glucose-dependent insulinotropic polypeptide; GLP-1, glucagon-like peptide-1.

IGR was increased throughout the MMTT in the *Hypo* group (iAUC: *p*=0.011, **Figure 4A**). Peak incretin/glucagon ratio, computed from GLP-1, GIP, and glucagon levels, was found to be significantly higher in *Hypo* group (30 min: *p*=0.008, **Figure 4B**).

## Discussion

This study provides insights into hormonal dynamics involved in PBH occurring as a late complication of interventions, as RYGB (2). During a provocative meal test, a positive correlation between early postprandial glucagon excursion and later glycemic nadir was identified, disclosing a potential role for glucagon in preventing hypoglycemia.

To conduct this study, subjects submitted to RYGB that spontaneously reported symptoms suggestive of hypoglycemia and asymptomatic controls underwent a mixed meal test. Our herein study was designed to assess the hormonal dynamics prompting hypoglycemia. Hence the test duration was set on 120 min, rather a longer duration that would be required to assess counterregulatory responses to hypoglycemia (29).

A glucose cut-off value of 3.05 mmol/l was selected to define hypoglycemia, in line with previous studies (10, 11, 15). In response to the meal stimulus, postprandial hypoglycemia occurred in a similar proportion in *Sym* and *Asy* groups, as previously reported (30). Thus, our findings further support that the occurrence of reactive hypoglycemia per se during a provocative meal test is not useful to diagnose PBH in patients presenting suggestive symptoms.

Diagnosing PBH poses several challenges that span from the clinical manifestations to diagnostic test selection and interpretation. The very first obstacle is symptoms under- or mis-reporting, due to partial overlap with dumping syndrome and/or hypoglycemia unawareness (31–33). While we have previously shown that self-reported symptoms compatible with PBH might be useful for PBH diagnosis when paired along concurrent flash glucose monitoring data, caution shall be taken when considering symptoms alone for PBH diagnosis (33). Our current data reinforces the limited value of symptom reports, as no correlation was observed between symptoms and the glucose profile prompted by a standardized meal stimulus. In the face of our findings, authors advise against establishing PBH diagnosis solely based on patient symptom reports.

Moreover, postprandial hypoglycemia is highly dependent on meal composition, size and texture (2, 13). So, despite the use of standardized macronutrient-balanced meal stimulus, there is no guaranty that a given meal will be able to replicate the conditions leading to PBH in each individual patient or provide clinically useful information.

After this initial observation, subjects were re-allocated according to glucose profile during the meal test into two new groups depending on the occurrence of postprandial hypoglycemia or not (*Hypo* and *NoHypo*). No differences in clinical features that could provide possible explanations for a distinct glucose meal test response to the same stimulus were found between subjects of *Hypo* and *NoHypo* groups. Then, the hormone dynamics that ultimately lead to postprandial hypoglycemia were analyzed.

There are several levels of evidence in support of the role of gut-hormone dynamics in triggering PBH (7). RYGB produces major modifications in gut anatomy that invariably increase the rate of intestinal nutrient exposure (34, 35) and occasionally interfere with vagus nerve integrity potentially compromising autonomic pancreatic and gastro-intestinal innervation (36, 37). In order to minimize the impact of anatomical diversity on gut hormone responses, only subjects that underwent a standardized procedure performed by the same surgical team were enrolled in this study (17). During the meal test, no significant differences in meal-triggered vasoactive response (pulse and BP, data not shown) were observed between subject groups. Hence, suggesting no major variations in gastric emptying rate although not formally assessed.

Nevertheless, subjects with meal-triggered hypoglycemic response had higher postprandial insulin and C-peptide excursions, without altered insulin sensitivity or beta-cell function, thus, reinforcing the role of hyperinsulinism, with peak levels 2-times higher, as the central mechanism of PBH (8, 10).

Furthermore, our results suggest that a postprandial glucagon excursion preceding insulin peak might increase glucose levels at nadir, thus preventing hypoglycemia. Indeed, glucagon was proposed to be involved in several physiological actions beyond the classical insulin counter regulatory actions namely mediating the liver-alfa cell axis (11, 38). Therefore, the hypothesis that early glucagon response “primes” the liver to maintain a sustained glucose production relevant at later time points after the meal stimulus cannot be excluded. Moreover, when insulin and glucagon levels were combined in the IGR, lower IGR (27) was observed in the group without hypoglycemia, suggesting a catabolic status with increased hepatic glucose output and further stressing hypothesis. In a previous study by Tharakan et al, higher glucagon levels 30 min after a meal were associated with higher rates of later postprandial hypoglycemia, which led the authors to propose that pancreatic glucagon might contribute to an exaggerated insulin response (11). However, in our study, an even earlier glucagon response 15 min after the meal was associated with lower rates of postprandial hypoglycemia, suggesting a protective role against hypoglycemia. The fact that the “protective” glucagon peak was observed 15 min after meal, while in the previous study glucagon levels were first evaluated only 30 min after the meal, could provide an explanation for the differences observed.

Still, despite this early glucagon excursion it should be noticed that a late phase glucagon response to a glucose lowering trend was also observed, along with insulin, C-peptide and incretins’ hormone suppression, in line with the well described glucagon counter-regulatory role (10, 38).

After RYGB, the exaggerated postprandial glucagon excursion in response to a mixed-meal was previously reported (11, 35, 39). However, the anatomical origin and mechanisms leading to postprandial glucagon excursion in these patients are not fully disclosed. In fact, glucagon secretion was expected to be suppressed by the simultaneous high glucose and GLP-1 levels (40), although not observed. Post-prandial glucagon could be secreted either by intestinal L-cells (38, 41) or by cephalic phase release from pancreatic alpha cells (42). Indeed, a subset of L-cells co-secreting GLP-1 and glucagon was identified in subjects after RYGB, but not before surgery (39). Overall, these factors could contribute to modify GLP-1 and glucagon secretion patterns, although these hypotheses require to be confirmed. The physiological stimulus for postprandial glucagon secretion is also a matter of debate. In addition to hypoglycemia, amino acids are known to play a pivotal role in glucagon secretion (38). After RYGB, earlier intestinal amino acid absorption is reported to lead to higher and more precocious postprandial amino acid plasma levels (43). Since no major differences in total amino acids postprandial profiles were observed between groups, our data does not support an amino acid contribution for the early glucagonemic response observed. Nevertheless, specific glucagonotropic amino acid levels, namely of alanine, tyrosine or glutamine, which might provide further insights into amino acids-glucagon dynamics, were not measured (44). Thus, despite total amino acid excursions did not provide evidence supporting a potential role for amino acids in prompting the differential glucagon excursions, it cannot be ruled out since the individual amino acids’ levels were not quantified.

The next question is what to expect from a higher glucagon response. Historically, according to the glucostatic theory of appetite control, one could predict that postprandial hyperglucagonemia observed after weight loss would not only prevent hypoglycemia and raise glycemic nadir, but also reduce hunger and suppress food intake, thus contributing to sustain weight loss (5, 6). However, PBH is usually associated with weight regain (45). Nevertheless, the potential use of glucagon for treating patients with PBH was tested using low-dose closed-loop infusion pumps that demonstrated to reduce the rates of hypoglycemia and prevent rebound hyperglycemia (16).

GLP-1 and GIP are incretin hormones with well-demonstrated insulinotropic effects (40). In the present study, to minimize the impact of active hormone levels interindividual variability derived from variable dipeptidyl peptidase 4 (DPP4) activity according to individuals’ weight and circulating insulin levels (46), total GLP-1 and GIP levels were measured. Subjects that developed postprandial hypoglycemia presented higher postprandial GLP-1 levels, consistent with some (2, 11) but not all previous reports (30).

The demonstration that blocking GLP-1 receptor with exendin 9-39 could mitigate the occurrence of postprandial hypoglycemia (10, 47) lead the authors to hypothesize that GLP-1 had a relevant role in mediating PBH. In contrast, in our study the finding that GLP-1 levels preceding insulin peak were similar in the two groups does not support a central role for GLP-1 in triggering the distinct hyperinsulinemic response. Moreover, no significant correlation between GLP-1 levels and later hypoglycemia was found. Similarly, no significant differences in fasting or postprandial GIP levels were observed between the groups to suggest a direct involvement in mediating PBH, a finding that is consistent with prior observations (11). Nevertheless, the GLP-1 insulinotropic potential is well-established and the possibility that a mismatch between different hormonal excursions and the timepoints considered cannot be ruled out and might sustain the similarities observed for postprandial excursions. Lastly, since GIP was demonstrated to induce glucagon secretion a potential contribution cannot be fully excluded nor confirmed (48).

In an attempt to infer the putative impact of the combined action of the different hormones that influence glucose dynamics, taking into account the GLP-1 and GIP insulinogenic action (40) and the counter regulatory effect of glucagon (38), an incretin/glucagon ratio was computed. This post-hoc exploratory analysis derived from the hypothesis that PBH is the end result of the unbalance between early endogenous glucagon insulin-antagonistic effect and insulinotropic stimuli. Indeed, our results support our hypothesis by revealing a higher incretin/glucagon ratio in those patients that develop postprandial hypoglycemia.

This study presents some limitations that must be acknowledged. This was an exploratory observational study which included a relatively small sample of patients submitted to RYGB at a single hospital institution, therefore limiting data extrapolations to other types of bariatric surgery interventions or mechanistic interpretations. Additionally, subjects had no dietary restrictions imposed on the days prior to the meal challenge, which could have influenced the meal response. Moreover, the larger time intervals between hormone assessments after the 60 min timepoints against the performed in early postprandial period, in addition to the 120 min for the total duration of the meal test precluded a more detailed evaluation of the hormone dynamics. The panel of hormones measured did not include counter regulatory hormones, such as cortisol and growth hormone, which could have limited the characterization of all endocrine pathways involved. Finally, the incretin/glucagon ratio herein computed for the first time with the rationale of assessing the balance between hormones known to influence postprandial glycemia, still requires further validation. Nevertheless, this study major strength is to provide insights into the early gut-pancreatic hormone dynamics associated with PBH, unravelling the importance of glucagon and incretin/glucagon balance, thus setting the grounds for further research over the molecular pathways leading to PBH.

Our study provides novel insights into the potential role of glucagon in preventing postprandial hypoglycemia, which may contribute to devising targeted medical or surgical interventions to prevent and manage PBH.

## Data Availability Statement

The raw data supporting the conclusions of this article will be made available by the authors upon justified request, without undue reservation.

## Ethics Statement

The studies involving human participants were reviewed and approved by Local Institutional Ethics Committee (Comissão de Ética para a Saúde CHEDV, Epe). The patients/participants provided their written informed consent to participate in this study.

## Author Contributions

CL, SP, MG, MN, and MM were responsible for study conception and design. CL, MG, SP, BH, NW, and LH performed experiments. CL and SP analyzed data. CL, SP, MG, MN, JH, and MM interpreted results of experiments. CL prepared figures and drafted the manuscript. SP, MG, BH, NW, LH, JH, MN, and MM edited and revised it critically for relevant intellectual content. All authors contributed to the article and approved the submitted version. MM is the guarantor of this work.

## Funding

Unit for Multidisciplinary Research in Biomedicine (UMIB) is funded by the Foundation for Science and Technology (FCT) Portugal (grant numbers UID/MULTI/0215/2016, UID/Multi/00215/2019, UIDB/00215/2020, and UIDP/00215/2020). JH holds an unrestricted grant from the Novo Nordisk Foundation Center for Basic Metabolic Research, Copenhagen, Denmark. The NNF Foundation Center for Basic Metabolic Research is an independent research institution at the University of Copenhagen, Denmark.

## Conflict of Interest

The authors declare that the research was conducted in the absence of any commercial or financial relationships that could be construed as a potential conflict of interest.
