# Supplementary material for: A Potential Role for Endogenous Glucagon in Preventing Post-Bariatric Hypoglycemia
Source: Front Endocrinol (Lausanne). 2020 Nov 30;11:608248. doi: 10.3389/fendo.2020.608248 (PMC7793799; doi:10.3389/fendo.2020.608248)
Supplement: Supplementary file 1 [file Table_1.pdf]

Supplementary Material

1 Supplementary Table 1

Anthropometric, demographic and metabolic features of post-RYGB patients according to clinical presentation (*Sym* and *Asy*).

	**Clinical presentation**		
	Sym	***Asy***	***p* value**
**N** (% of total)	14 (60.9%)	9 (39.1%)	NA
**Sex** (male/female)	3/11	1/8	1.000
**Age at surgery** (years)	44.5 ± 2.2	38.1 ± 3.0	0.097
**History of T2DM before surgery** (yes/no)	3/11	0/9	0.253
**Follow-up time after surgery** (years)	4.8 ± 0.6	3.9 ± 0.7	0.318
**BMI before surgery** (kg/m^2)	40.2 ± 1.6	41.8 ± 1.2	0.472
**BMI after surgery**(kg/m^2)	28.0 ± 0.9	28.1 ± 0.8	0.952
**%EBMIL** (%)	83.1 ± 5.2	81.7 ± 4.8	0.860
**%TWL** (%)	29.7 ± 1.9	32.5 ± 2.1	0.352
**HbA1c** (mmol/mol)	35.1 ± 1.0	35.1 ± 1.4	1.000
**HbA1c** (%)	5.4 ± 0.1	5.4 ± 0.1	1.000
**HOMA2-B** (%)	68.5 ± 6.2	82.0 ± 4.5	0.129
**HOMA2-S** (%)	160.0 ± 12.0	130.4 ± 14.9	0.138
**HOMA2-IR**	0.6 (0.5-0.8)	0.8 (0.6-1.0)	0.154
**Glycaemic nadir MMTT <3.05mmol/l** (yes/no)	7/7	4/5	1.000
**Dumping criteria** (yes/no)	13/1	8/1	0.478

Results are presented as mean ± SEM, median (interquartile range) or proportions. Abbreviations: MMTT – mixed-meal tolerance test; *Sym* – Subjects reporting symptomatology compatible with post-bariatric hypoglycemia; *Asy* – Asymptomatic surgical controls; T2DM – type 2 diabetes mellitus; BMI – body mass index; EBMIL – excess BMI loss; TWL – total weight loss; HOMA2-B - homeostasis model assessment for β-cell function; HOMA2-S - homeostasis model assessment for insulin sensitivity; HOMA2-IR – homeostasis model assessment for insulin resistance.
